# Supplementary material for: Knee Joint Contact Forces during High-Risk Dynamic Tasks: 90° Change of Direction and Deceleration Movements
Source: Bioengineering (Basel). 2023 Jan 31;10(2):179. doi: 10.3390/bioengineering10020179 (PMC9952676; doi:10.3390/bioengineering10020179)
Supplement: Supplementary file 1 [file bioengineering-10-00179-s001.zip › bioengineering-2188333-supplementary.pdf]

Supplementary material

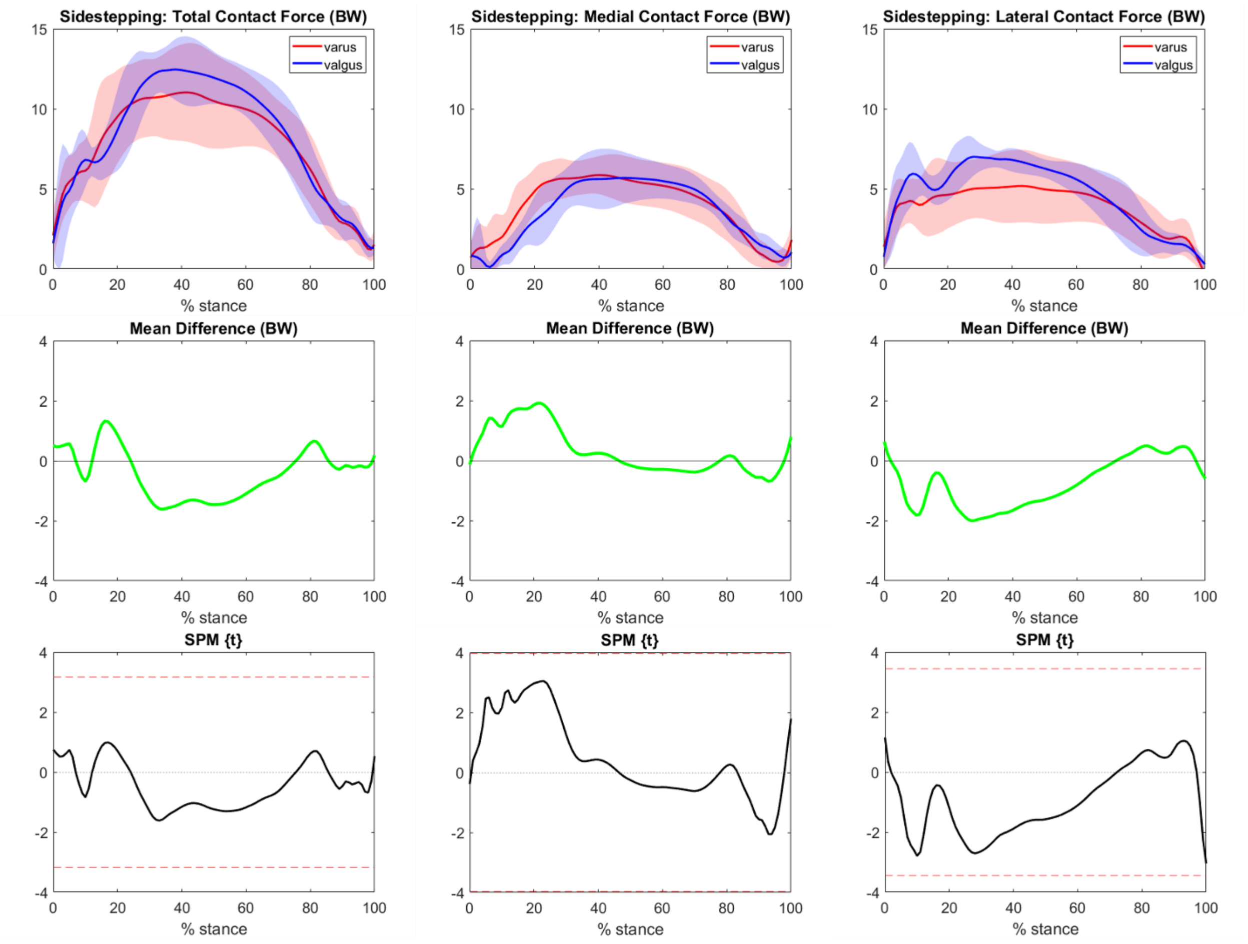

Figure S1: Sidestepping TFCF (normalized to body weight and represented as mean and standard deviation) across the task stance, compared between varus (red) and valgus (blue) groups. Left column represents Total TFCF, middle column represents Medial TFCF, and right column represents Lateral TFCF. The central row highlights the differences between the means (varus-valgus). Grey areas with corresponding *p*-values from SPM graphs indicate significant and clinically relevant differences.

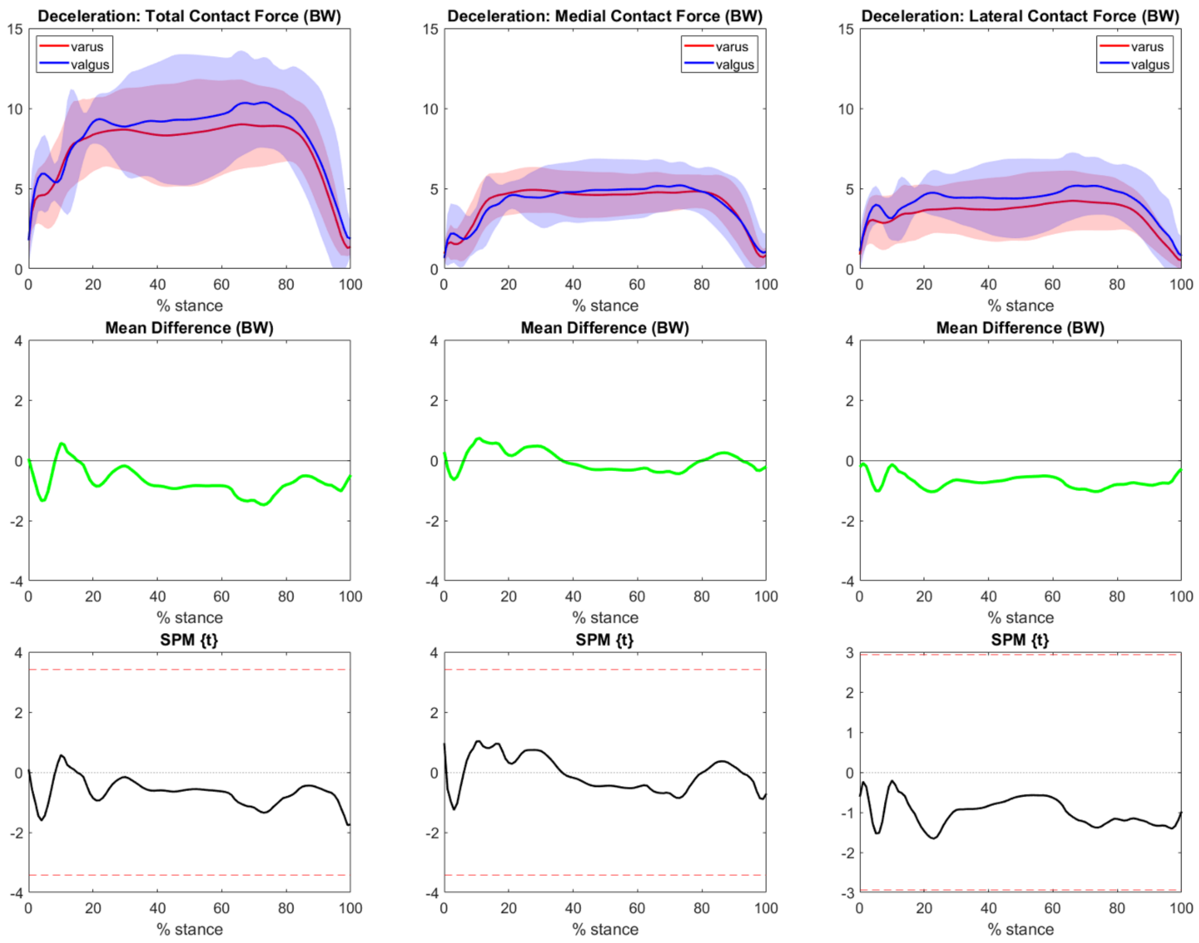

Figure S2: Deceleration TFCF (normalized to body weight and represented as mean and standard deviation) across the task stance, compared between varus (red) and valgus (blue) groups. Left column represents Total TFCF, middle column represents Medial TFCF, and right column represents Lateral TFCF. The central row highlights the differences between the means (varus-valgus). Grey areas with corresponding *p*-values from SPM graphs indicate significant and clinically relevant differences.

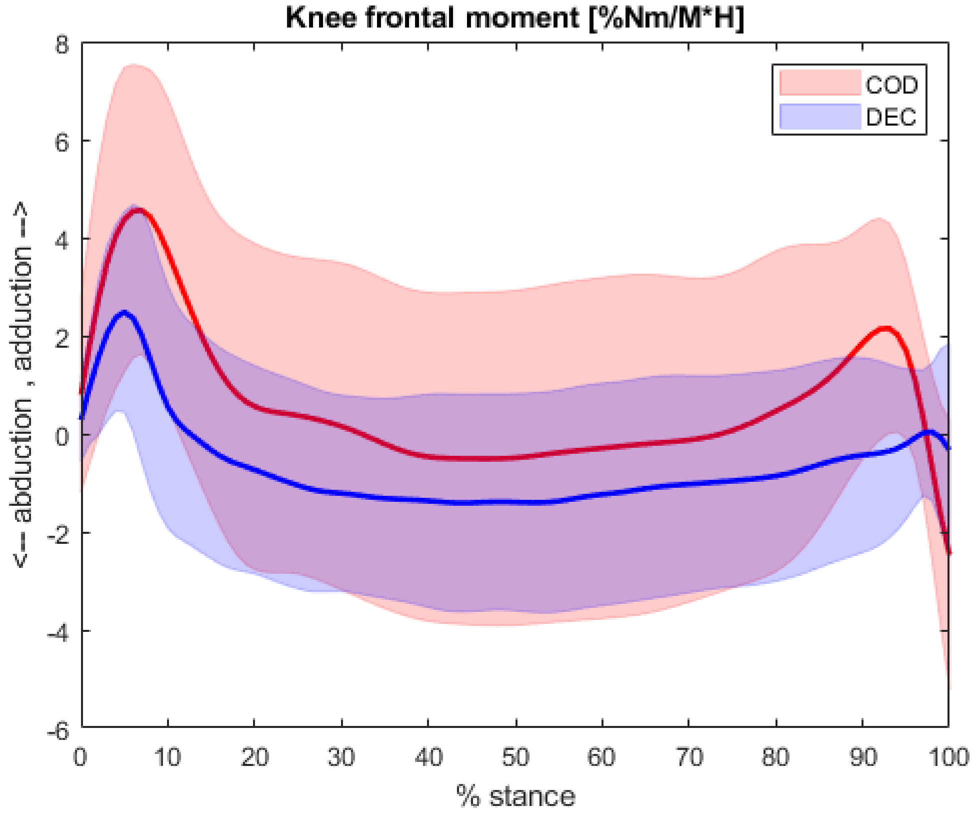

Figure S3: Knee frontal reaction moment during COD (red) and DEC (blue) estimated by simulations. Values were divided by body weight (M) and total height (H). At the beginning of both tasks an external abduction moment generated an augmentation of lateral tibiofemoral contact forces. For COD task, also the final part before toe-off was characterized by the presence of an external abduction moment and subsequent rise in lateral tibiofemoral contact forces.
